# Are the medio-lateral joint forces in the lower limbs different between scoliotic and healthy subjects during gait?

**DOI:** 10.1186/1748-7161-10-S1-O30

**Published:** 2015-01-19

**Authors:** Mouna Yazji, Maxime Raison, Carl-Éric Aubin, Hubert Labelle, Christine Detrembleur, Philippe Mahaudens, Mousny Marilyne

**Affiliations:** 1 Polytechnique Montreal, Canada; 2 Research Center of Sainte-Justine UHC, Montreal, Canada; 3 Institute of Neurosciences (IoNS), Universite Catholique de Louvain (UCL), Bruxelles, Belgium; 4 Service d'Orthopedie et de Traumatologie de l'appareil locomoteur (ORTO), Saint-Luc UHC, Brussels, Belgium

## Objective

To compare the medio-lateral joint forces in the lower limbs during gait between healthy subjects and patients with different scoliotic severities, to develop indicators of quality and comfort during gait.

## Material and methods

Twelve healthy subjects, 12 adolescents with idiopathic scoliosis (AIS) with Cobb angle (CA) between 20 and 40 and 16 AIS in pre-operative condition (Cobb : > 40) performed gait at 4 km/h on an instrumented treadmill. The acquisition system included optokinetic sensors (BTS, Italy) recording the 3D joint coordinates and a treadmill equipped with force sensors (UCL, Belgium) measuring the 3D external forces independently applied to each feet. The hip, knee, and ankle joint forces at the left and right lower limbs were calculated using a tridimensional inverse dynamic model of the human body. One-way ANOVA was performed for the maximum, the minimum and the range of medio-lateral forces at each joint of the lower limbs. When appropriate, a Tukey's post hoc was performed to determine the differences.

## Results

The maxima and the magnitudes of the medio-lateral forces at the right hips and knees were significantly lower in AIS having a CA between 20 and 40 compared to healthy subjects. At both left and right ankle joints, the medio-lateral forces showed significant differences between healthy subjects and the two AIS subgroups.

## Conclusion

The medio-lateral joint forces in the lower limbs are significantly different between AIS and healthy subjects. The spinal deformity correlates to a decrease of hip, knee, and ankle medio-lateral forces. This force decrease can be explained by the reduced muscle efficiency that results from the longer contraction time of the lumbar and pelvic muscles. This force decrease could gradually change the pattern of postural adjustments in AIS during gait. Especially at the hip, the decreased medio-lateral force corresponds to a postural adjustment balancing the increased pelvic moment generated by the medio-lateral shift of the thoracic mass during gait. Consequently, the evaluation of the medio-lateral forces in the lower limbs could help to select specific postural rehabilitation exercises around each lower limb joint to develop indicators of quality and comfort during gait.

